# Tuning of the Na,K-ATPase by the beta subunit

**DOI:** 10.1038/srep20442

**Published:** 2016-02-05

**Authors:** Florian Hilbers, Wojciech Kopec, Toke Jost Isaksen, Thomas Hellesøe Holm, Karin Lykke-Hartmann, Poul Nissen, Himanshu Khandelia, Hanne Poulsen

**Affiliations:** 1 Danish Research Institute of Translational Neuroscience – DANDRITE, Nordic EMBL Partnership for Molecular Medicine, Aarhus University, DK-8000 Aarhus, Denmark; 2 Department of Molecular Biology and Genetics, Aarhus University, DK-8000 Aarhus, Denmark; 3 Centre for Membrane Pumps in Cells and Disease – PUMPKIN, Danish National Research Foundation, DK-8000 Aarhus, Denmark; 4 MEMPHYS: Centre for Biomembrane Physics, University of Southern Denmark, DK-8000 Aarhus, Denmark; 5 Department of Biomedicine, Aarhus University, DK-8000 Aarhus, Denmark; 6 Aarhus Institute of Advanced Studies (AIAS), Aarhus University, DK-8000 Aarhus, Denmark

## Abstract

The vital gradients of Na^+^ and K^+^ across the plasma membrane of animal cells are maintained by the Na,K-ATPase, an αβ enzyme complex, whose α subunit carries out the ion transport and ATP hydrolysis. The specific roles of the β subunit isoforms are less clear, though β2 is essential for motor physiology in mammals. Here, we show that compared to β1 and β3, β2 stabilizes the Na^+^-occluded E1P state relative to the outward-open E2P state, and that the effect is mediated by its transmembrane domain. Molecular dynamics simulations further demonstrate that the tilt angle of the β transmembrane helix correlates with its functional effect, suggesting that the relative orientation of β modulates ion binding at the α subunit. β2 is primarily expressed in granule neurons and glomeruli in the cerebellum, and we propose that its unique functional characteristics are important to respond appropriately to the cerebellar Na^+^ and K^+^ gradients.

The Na,K-ATPase (NaKA) transports three Na^+^ from inside the cell to the outside coupled to auto-phosphorylation from ATP and two K^+^ from outside to inside coupled to auto-dephosphorylation in a reaction cycle where the conformations with high Na^+^ affinity are termed E1 and those with high K^+^ affinity are termed E2 (Fig. 1a)^1^,^2^,^3^,^4^,^5^,^6^. The ATP-driven reaction proceeds against the concentration gradients of both ions and generates steep electrochemical gradients across the plasma membrane that are used for a variety of cellular processes including neuronal signalling and secondary active transport^7^,^8^. The 3:2 stoichiometry of ion transport means that the activity of the pump can be determined from the steady-state current it generates, and under restricting conditions, individual voltage-sensitive steps in the catalytic cycle can be monitored. The extracellular translocation of each of the three Na^+^ is voltage-dependent, and the relatively slow pre-steady-state charge movement associated with the third Na^+^ can readily be recorded if the NaKA is restricted to binding and releasing Na^+^ by omission of extracellelular K^+^^9^,^10^. The pre-steady-state currents reflect the voltage-dependent E1P-E2P transition (Fig. 1a)^9^, where the probability of the NaKA being in the Na^+^-occluded E1P state is highest at negative membrane potentials and in the outward-open E2P state highest at positive membrane potentials.

The minimal pump has two subunits, α and β, and can further interact with a γ (FXYD) subunit. Humans express four isoforms of α, three of β, and seven of FXYD^11^, while insects have a single functional α subunit and several β subunits. Deletion or mutation of the β subunit can have severe consequences. In *Drosophila*, the β subunits regulate sight and hearing^12^, and in mice, deletion of the gene encoding β2 gene causes motor disabilities, and the animals die a few weeks after birth^13^. In humans, changes in the expression pattern of β2 have been linked to glioma^14^.

Different α/β combinations were previously shown to have different apparent K^+^ affinities^15^, especially α2β2 has very low apparent K^+^ affinity, but a high turn-over-rate, and is suggested to be specifically geared for K^+^-clearance in hippocampal glia cells^16^ and in fast-twitch glycotic muscle fibers^17^,^18^. In brain, the expression profile for β2-encoding mRNA indicates high expression in cerebellum^19^, and protein stainings show β2 in Purkinje cells with α2, α3 and β1, and in granule cells and glomeruli with α1, α3 and β1^20^. In cerebellum, about 60% of the ATP consumption is estimated to be used to maintain the ionic gradients required for signalling, and almost 70% of that ATP is used by the granule cells^21^, so the NaKA activity in granule cells is clearly a dominant factor in the overall energy consumption in the cerebellar cortex.

We have investigated the molecular and functional role of β2 and find that it significantly influences the E1P-E2P equilibrium with any of the α subunits studied. To determine the molecular mechanism of β’s functional effect, we constructed chimeras of β1 and β2, which pinpointed the transmembrane domain as the main determinant for the observed electrophysiological characteristics. Molecular dynamics (MD) simulations suggest that the transmembrane helices of β1 and β2 have different tilt angles, and we propose that the tilt angle of β can influence the relative stability of the Na^+^ occluded E1P state.

## Results

### The β2 subunit is highly expressed in mouse cerebellum

The α2β2 combination has been suggested to be important for K^+^ clearing in hippocampus and fast-twitch muscles^16^,^18^, but β2’s role in motor coordination^13^ implies an important role of its high expression in cerebellum^19^. To determine the relative levels of β2 expression in the brain, we performed Western blot analysis of isolated regions of mouse brain. We found the highest expression of the β2 isoform in cerebellum (Fig. 2a), and to further delineate, which cells express β2, brain slices of adult three months old mice were immunostained. There was intense staining for β2 in NeuN-positive granule neurons^22^ and at glomeruli in the GCL (Fig. 2c), while the Purkinje cell layer had diffuse staining with no signals in the Purkinje cell bodies and only weak, punctuated staining in the molecular layer (ML).

We similarly stained for β1 and observed it in the NeuN-positive granule neuron plasma membrane and at glomeruli in GCL as well as in the cell bodies and pinceau of Purkinje cells (Fig. 2b). In the ML, fibers and cell bodies showed punctuated β1 staining. Staining for the α1 subunit also showed expression in granule neurons and at glomeruli (Supplementary Fig. S1), suggesting that both α1β1 and α1β2 pumps may form in the GCL.

Like cerebellum, the dentate gyrus of hippocampus has high neuronal density. To examine if β2 expression is a general phenomenon in regions with high neuronal density, we examined the dentate gyrus GCL. However, no β2 staining was observed, and only modest, punctate staining was seen in the ML and Hilus, while both β1 and α1 clearly stained the GCL, ML and Hilus in the dentate gyrus (Supplementary Fig. S1).

### Pumps with β2 have high affinity for extracellular Na^+^

**Figure 1:**
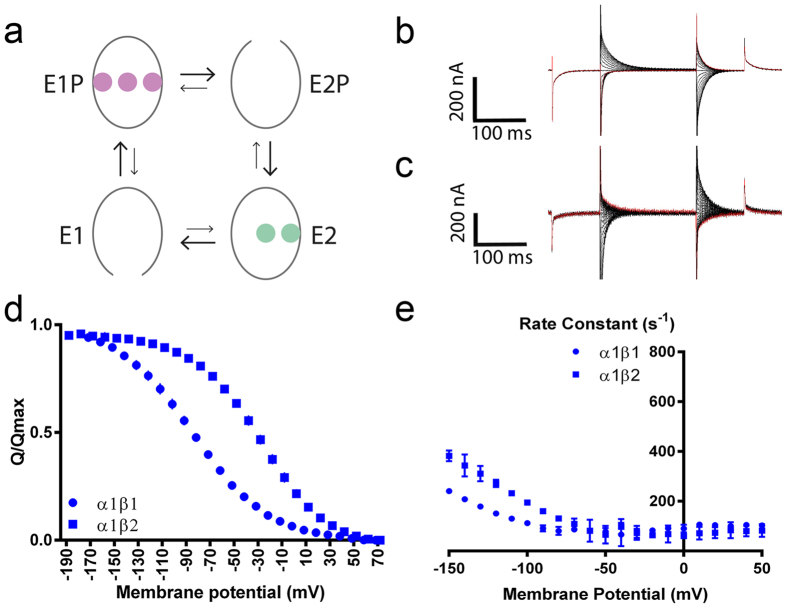
Electrophysiological properties of α1β1 and α1β2. A simplified Post-Albers scheme with Na^+^ in purple and K^+^ in green is shown in (**a**). Difference curves in K^+^-free buffer with and without 10 mM ouabain for (**b**) α1β1,(**c**) α1β2 are shown. The curves were fitted with single exponentials, giving the voltage dependent (**d**) charge translocation from the off currents and (**e**) rate constants from the on currents. N = 3–10 with oocytes from at least two *Xenopus laevis* females. Data are represented as mean ± SD.

To delineate the functional difference between α1β1 and α1β2, we expressed the pumps in oocytes from *Xenopus laevis* and determined their electrophysiological characteristics with two-electrode voltage clamping (TEVC). In the absence of extracellular K^+^, the NaKA is restricted to binding and releasing Na^+^ from the extracellular medium^9^,^10^, and the relatively slow charge movement associated with the third Na^+^ can readily be recorded (Fig. 1). The charge translocation in response to a change in the membrane potential (Q/V curve) reflects the voltage-dependent E1P-E2P transition^9^, and right-shifting of the Q/V curve signifies a relative stabilization of the E1P state.

**Figure 2:**
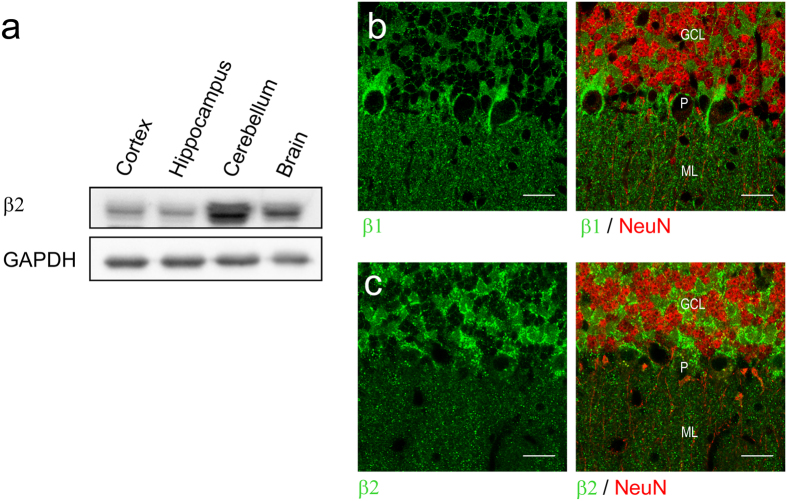
Expression and localization of the β1 and β2 isoforms in mouse brain. (**a**) Western blot analysis of the indicated isolated mouse brain regions using antibodies against β2 and GAPDH. (**b**) Fluorescence immunohistochemistry of β1 and β2 in cerebellum co-stained with the neuronal marker NeuN. GCL: granule cell layer. P: Purkinje cell layer. ML: molecular layer. Scale bars represent 20 μm.

Compared to α1β1, α1β2 has a right-shifted Q/V curve (midpoint potentials V_0.5_ –82.8 mV ± 0.6 mV with β1, –29.4 mV ± 0.4 mV with β2; Fig. 2d; Supplementary Table S1), and α1β2 has higher rate constants than α1β1 at hyperpolarized potentials (Fig. 1e).

It is well known that mutations and subunit differences in the α subunit can influence the midpoint potential^23^,^24^. To examine if the β2 effect is specific for α1, we therefore expressed the various combinations of α1, α2 and α3 with β1, β2 and β3 and determined their V_0.5_ and rate constants. We found that with any given α subunit, pumps with β2 have right-shifted Q/V curves and higher rate constants compared to pumps with β1 or β3 (Supplementary Fig. S2 and Supplementary Table S1).

### Voltage dependence of maximal turnover is similar with different β subunits

At hyperpolarized potentials, α2β2 has much lower apparent K^+^ affinity than the other combinations of α1, α2 or α3 with β1 or β2^15^,^16^. To estimate the voltage dependence at maximal turnover, we determined the steady-state currents with 15 mM K^+^ in the extracellular buffer. No significant effect of β is evident for the α1 or α2 combinations (Supplementary Fig. S3a-c). With sub-saturating K^+^-concentrations, the α2β2 pumping has previously been reported to have a stronger voltage-dependence than the other combinations^15^,^16^, suggesting that the main determinant is the voltage-sensitive K^+^-affinity, which may partly reflect its stronger E1P preference. In the absence of extracellular Na^+^ and K^+^, NaKA carries an inwardly rectifying proton current^25^,^26^. The inward proton currents were similar although slightly larger for α1β2 (Supplementary Fig. S3d). Omission of extracellular Na^+^ and K^+^ has also been associated with an uncoupled Na^+^ efflux^27^, but this reaction is orders of magnitude slower than the regular pumping^28^, while the proton current can be even larger than the forward pumping current at hyperpolarized membrane potentials (Supplementary Fig. S3d).

### The effect of the β subunit is determined by its transmembrane helix

**Figure 3:**
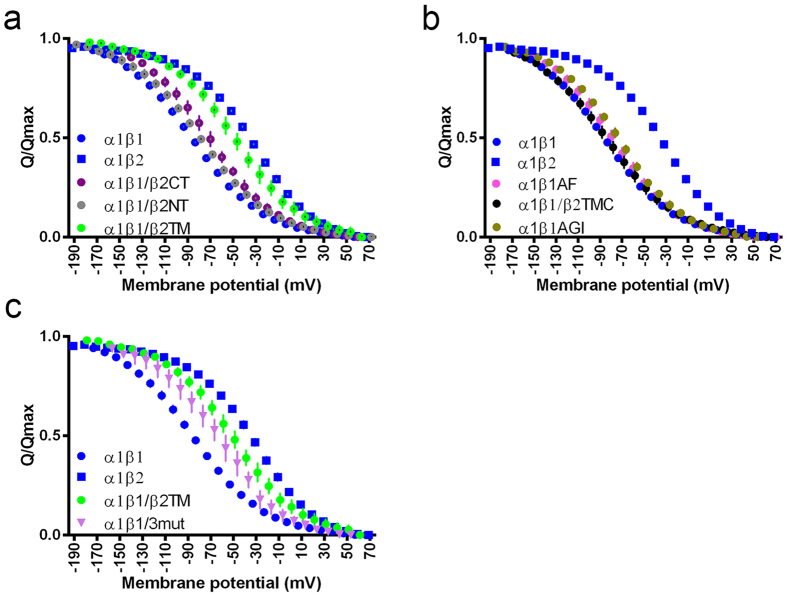
Charge translocation curves of chimeras and pocket mutants. Charge translocation was determined for α1 coexpressed with (**a**) β chimeras, where the N-terminal, the C-terminal or the transmembrane region of β1 was replaced with that of β2, with (**b**) β mutants where smaller stretches in the transmembrane region of β1 were replaced with the corresponding β2 sequences, FK with AF N-terminally, AGI with TAM in the middle or the C-terminal 16 residues, or (**c**) a combination of the FK toAF , AGI to TAM and VSD to ISE (at the C-terminus, cf. Fig. S6) in β1 giving β1/3mut. N ≥ 5 with oocytes from at least two *Xenopus laevis* females. Data are represented as mean ± SD.

Next, we asked if we could map the region of β2 that markedly influences the E1P-E2P equilibrium. We constructed three chimeras of β1 and β2, replacing either the N-terminal cytoplasmic domain, the transmembrane domain or the C-terminal extracellular domain of β1 with the corresponding β2 sequence, giving β1/β2 NT, β1/β2TM and β1/β2CT, respectively (Fig. 3a).

The Q/V curve for β1/β2NT (V_50_ –80 mV) is very close to that of β1 (V_50_ – 83 mV), and β1/β2CT is also only slightly shifted (V_50_ –70 mV). In contrast, β1/β2TM (V_50_ –51 mV) is closer to β2 (V_50_ –29 mV) than to β1 (Fig. 3a and Supplementary Table S1). This suggests that the main determinant of β’s effect on the E1P-E2P equilibrium is the transmembrane domain with a small, additive contribution from the large extracellular C-terminal domain. No significant differences in steady state currents with 15 mM K^+^ were observed between wild type and chimeras (Supplementary Fig. S3).

In the β transmembrane domain, the N-terminal part of the helix (towards the cytoplasmic side) has the highest degree of sequence conservation (Supplementary Fig. S4), so the more divergent C-terminal part of the helix in β1 was replaced by the β2 sequence. Surprisingly, the resulting chimera, β1/β2TMC (β1 with the β2 transmembrane C-terminus) had a Q/V curve similar to that of β1 (Fig. 3a). We therefore fine-tuned the mutational approach and introduced the most prominent differences between β1 and β2 in the remaining part of the helix into β1, changing an AGI motif to TAM in the middle (residues 47–49 in β1 giving β1TAM) and FK to AF towards the intracellular interface (residues 33–34 in β1giving β1AF) (Supplementary Fig. S4). The charge translocation curve of β1AF and β1TAM were only shifted 5 and 7 mV, respectively, towards β2 (Fig. 3b), changes too modest to explain the effect of the β transmembrane domain on the pump properties. However, combining the two mutations and a motif at the extracellular side, ISE to VSD (residues 61–63 in β1), in the mutant β1/3mut, gave a charge translocation curve that was shifted 18 mV towards β2 (Fig. 3c). This synergistic effect of the three mutated areas suggests an overall structural difference between β1 and β2 at the interface with α in the membrane.

### Molecular Dynamics simulations suggest different tilt angles of β1 and β2

**Figure 4:**
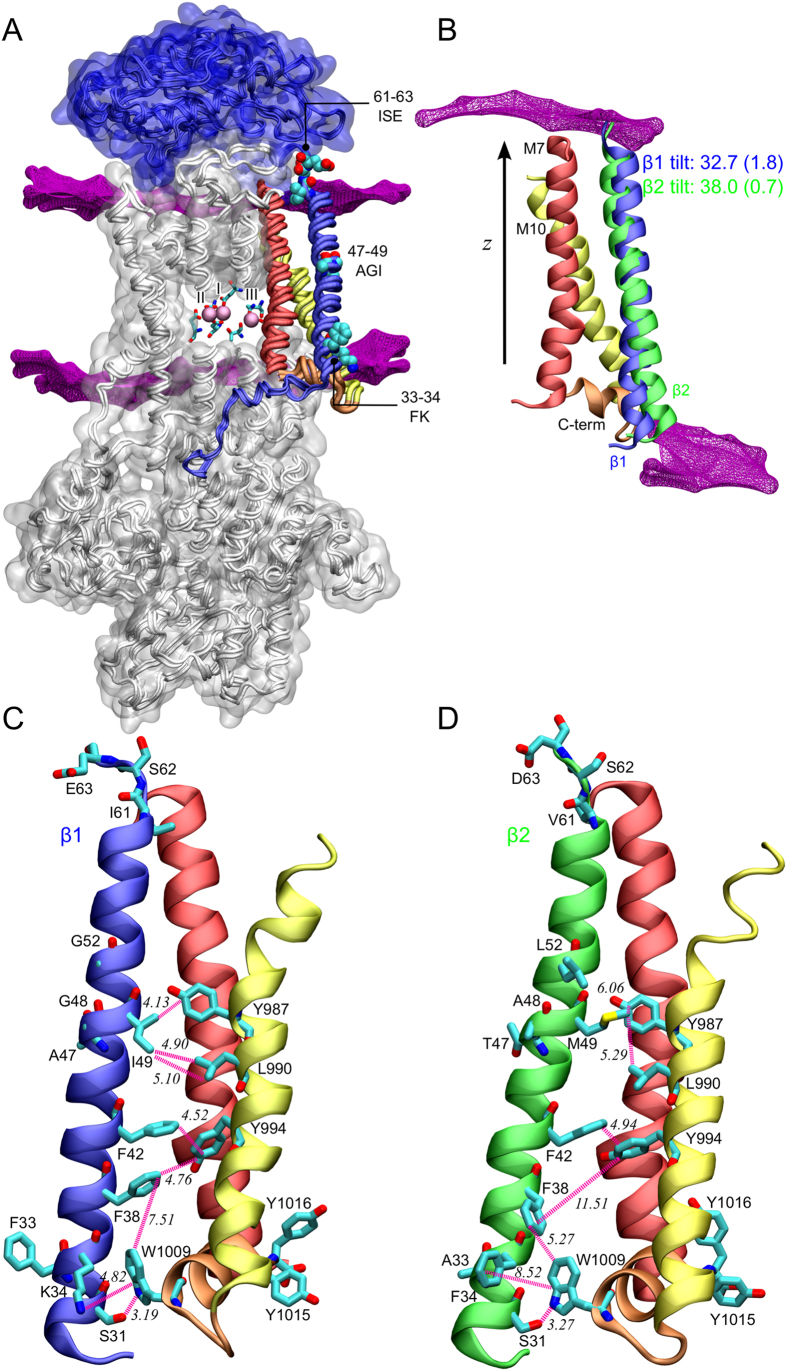
Molecular dynamics simulations of α1β1 and α1β2. (**a**) Atomistic models of the α (white-grey) and β1 (blue) subunits, embedded in the POPC lipid membrane (contour shown in magenta). Several important residues of the β subunit are shown in spacefill. Note that 33–34 FK and 61–63 ISE residues are located at or near the membrane interface. Important residues forming ion binding sites I, II and III are shown as sticks, and bound sodium ions are shown as pink spheres. Water and the γ subunit are omitted for clarity, but included in the model. (**b**) Comparison of the helix tilt between the transmembrane helix of β1 (blue) and β2 (light green). The tilt is defined as the angle between the helix axis and the z-axis, which is perpendicular to the membrane surface. Presented values are the averages from the last 40 ns, with error estimations obtained with block averaging. (**c**,**d**) Interaction patterns between the β helix (blue β1 C) and light green β2 D)) and the M7 helix (yellow) and the C-terminus (orange) of the α subunit in α1β1 C) and α1β2 D). Interactions between selected residues (shown in licorice) are shown as purple springs, with minimum distances recorded in simulations (last 40 ns) indicated in italics. Hydrophobic carbon atoms are shown in cyan, oxygen atoms bearing partial negative charge are shown in red and nitrogen atoms bearing partial positive charge are shown in deep blue.

To examine the interactions between the α and β subunits further, we analysed crystal structures and compared MD simulations of α1β1, α1β2 and α1β3. The crystal structure of the pig α1β1γ^4^ in the E1P state was used as the starting model, since it has the most complete structure of β, including the cytoplasmic domain, which is unresolved in other structures. α1β2 and α1β3 structures were constructed with homology models replacing β1 (Fig. 4 and Supplementary Fig. S5).

Guided by the electrophysiological measurements, we focused our analyses on the transmembrane region of the β subunits and their interactions with α. The interaction sites identified in earlier mutational studies (e.g. β1-Y39 with α1-S844 and β1-Y43 with α1-G848)^2^,^3^ are conserved in the βs^2^,^3^. However, we noticed a marked difference between subunits in tilt angle of the transmembrane helix, which was 32.7° ± 1.8° for α1β1 (in accordance with a helix tilt of 31.0° in the crystal structure) and 31.3° ± 0.9° for α1β3, but 38.0° ± 0.7° for α1β2 (Fig. 4, Supplementary Fig. S5 and table S1).

Comparing β1 and β2 shows that the different tilt angles manifest differences in interaction patterns between α and β, especially between the β helix and the α M10 helix (Fig. 4c,d) For example, the β1-AGI / β2-TAM motif, which had a small but measurable effect in electrophysiology, shows differences in interactions with the α M10: there are hydrophobic interactions between the isoleucine in β1 and residues in the α M10 approximately 10 Å from ion binding site III (Fig. 4c), but not between the longer methionine in β2 and the α residues (Fig. 4d).

**Figure 5:**
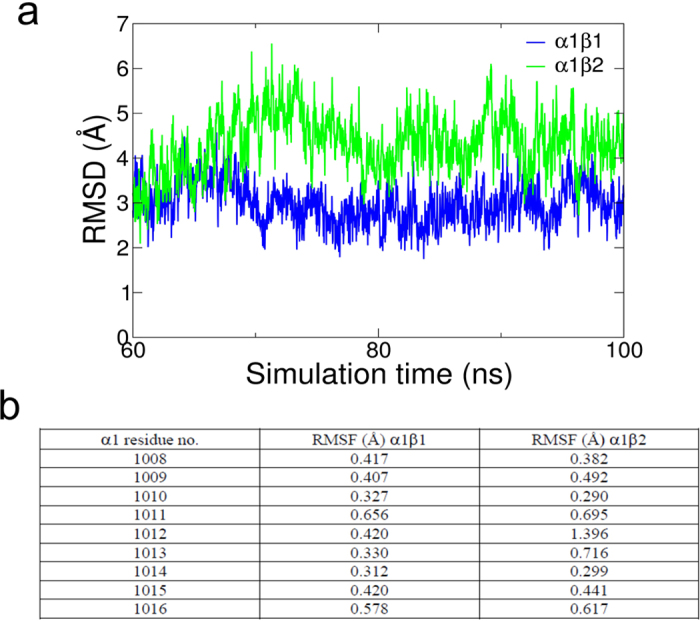
Flexibility of the α C-terminus with β1 or β2. (**a**) Root mean square deviation (RMSD) of the C-terminus of α1 with β1 or β2 showing displacement in α1β2. (**b**) Root mean square fluctuations (RMSF) of heavy atom positions of the protein residues that form the C-terminal tail of α1, average of the last 60 ns of the MD trajectory.

Interestingly, β1 and β2 appear to interact differently with the α C-terminus, which is known to be an important regulator of the Na^+^ binding site III^1^,^9^,^29^,^30^. A cation-π interaction between β1 K34 and α1 W1009 is absent with β2, which has an F at this position (Fig. 4C,D). A K in β1 and an F in β2 are conserved between species (human, rat, sheep, chicken and dolphin). With β2, the α1 C-terminus is consequently slightly displaced in the MD simulations, and there is higher fluctuation in the hinge region connecting it to M10 (Fig. 5), suggesting that sequence differences in the β subunits can affect the structure and flexibility of the α C-terminus and hence the E1P-E2P equilibrium.

The electrophysiological data suggest that single regions in the β transmembrane region have little or no effect, but combining mutations gives a pronounced shift (Fig. 3). If the subunit differences depend on the membrane tilt angle as the MD simulations imply, we would expect the transmembrane helices to interact differently with the lipid head groups. From the MD simulations, we calculated radial distribution functions (RDF) between the lipid headgroups and the expected anchoring points of the transmembrane helix (Supplementary Fig. S6). With β1, the helix is anchored on the intracellular side at the FK motif and at the extracellular side at the ISE motif (Supplementary Fig. S4). With β2, the lipid interactions are weaker at the intracellular side and stronger at the extracellular side, which likely contributes to the change in the tilt angle and thus to altered functional properties, strengthening the electrophysiological finding that a change in both membrane anchor points together with the AGI to TAM mutation in the middle changes β1 towards β2.

To test this hypothesis, we performed MD simulations of the triple mutant α1β1/3mut and of the transmembrane chimera α1β1/β2TM and found tilt angles of 36.0° ± 0.8° and 37.2° ± 0.7°, respectively (Supplementary Fig. S5). This fits well with the electrophysiological observation that the E1P-E2P equilibria of α1β1/3mut and α1β1/β2TM are shifted towards that of α1β2.

## Discussion

The unique properties of α2β2 have led to the suggestion that NaKA with this subunit composition is optimized for clearing of K^+^ from the extracellular fluid in hippocampal glia cells^16^ and in fast-twitch glycotic muscles^17^,^18^. The severe motor phenotype of mice lacking β2^13^ is likely to be due to impairment in the cerebellum, a region central to neuromuscular processing, where high levels of β2 are detected in rat^20^ and mouse (Fig. 2). The cerebellum has the highest ratio of neurons to glia cells in the brain, an estimated 4.3^31^, and about 40–50% of the cerebellar ATP is used to fuel NaKA in the granule cells^21^, where we observe intense β2 staining (Fig. 2c), suggesting that K^+^ clearance in cerebellar neurons may rely on β2 containing NaKA. Unlike a previous study^20^ , we did not find β2 expression in Purkinje cells.

Specific subunit localization may be due to developmental or targeting requirements, but our findings indicate that the functional characteristics of β2 are also likely to be important. We therefore focussed on determining the functional differences between β1 and β2. In the P-type ATPase family, the NaKA, H,K-ATPase (HKA) and the lipid flippases are unique in their strict dependence on a β subunit for trafficking of the holoenzyme to the plasmamembrane and for modulation of the catalytic properties. Combining the NaKA α with different β subunit isoforms or the β subunit from the HKA has previously shown that β affects the apparent K^+^ and Na^+^ affinities and changes the rate of formation of the phosphoenzyme^15^,^16^,^32^,^33^,^34^,^35^,^36^,^37^,^38^,^39^,^40^,^41^,^42^,^43^, while surface expression, turnover number and ouabain binding were similar for different β isoform combinations^15^.

NaKAs with β2 have shown the most significant differences with an unusually high K_1/2_ for K^+^ activation of α2β2 at hyperpolarized potentials^16^. Our data similarly imply that NaKA β2 combinations form the most divergent pumps. We show that, compared to β1 and β3, β2 markedly shifts the E1P-E2P equilibrium towards the E1P state (right-shift of the Q/V curve) (Fig. 1 and Supplementary Fig. S2) and increases the rate constants at hyperpolarized potentials (Supplementary Fig. S2).

To understand the mechanism of the functional effects of the β isoforms, we constructed chimeras of β1 and β2. Both the N-terminal cytoplasmic region and the C-terminal extracellular region of β1 could be exchanged for the corresponding β2 region without major effect on the Q/V curve’s midpoint potential. In contrast, replacing the transmembrane domain in β1 with that of β2 markedly shifted the Q/V curve towards that of β2 (Fig. 1d).

The β transmembrane domain was previously suggested to be involved in retention of the holoenzyme in the endoplasmatic reticulum, since chimeras of β1 with the transmembrane region of the HKA β are retained^34^. Furthermore, glutathionylation of a cysteine in the middle of β1’s transmembrane region was shown to influence the catalytic properties of the pump^44^, but the cysteine is absent from both β2 and β3 (Supplementary Fig. S4), and β3 behaves largely like β1 (Supplementary Fig. S2), so a difference in glutathionylation is unlikely to explain why β2 differs from the other βs.

The transmembrane domain of the β subunit is not highly conserved (Supplementary Fig. S4), and one or more of the 17 residues that differ between the β1 and β2 helices must account for the functional differences, but mutational studies did not identify any single motif as a key determinant (Fig. 3a,b). However, a significant shift of the charge translocation curve towards α1β2 was seen when combining mutations in three areas, at the N- and C-terminal membrane anchor points and in a central motif, suggesting that the tilt angle of the transmembrane helix may be important for the functional effects (Fig. 3c).

To examine the structural foundation for the differences, we performed MD simulations of α1β1 and of α1β2 and α1β3 homology models. The most striking difference between the structures was the β tilt angle relative to the membrane plane with β1 and β3 being similar, while β2 was 5° more tilted than β1, which changes e.g. the interactions between the α M10 and the highly conserved β F38 and between the α C-terminus and the AF/FK motif of β1 and β2 (Fig. 4). Thus, we find that the β isoforms can influence structural elements in the α subunit that are known to be important for the kinetic properties of Na^+^ binding^29^,^45^,^46^.

From the related calcium pump SERCA, several structures of different functional states have been determined, which indicate that the M7, M8, M9 and M10 move in concert as a C-terminal domain^47^,^48^,^49^. In NaKA, the corresponding C-terminal domain of α is important for ion binding and release at the unique site III^5^,^29^. There are only structures of a few functional NaKA states, but an overlay of the structures of the potassium-bound E2Pi-like state^2^ and the sodium-bound E1P-like state^4^,^5^ shows that the M10-β1 interface, including the β1 tilt angle, is conserved. The β helix thus appears to move together with the C-terminal helix bundle of α during the catalytic cycle, but the differences described here are likely to affect the overall dynamics of the bundle and thereby the properties of Na^+^ binding at site III.

In summary, the electrophysiological studies and MD simulations presented here indicate that a main determinant of the functional differences between β1 and β2 is the tilt angle of the transmembrane helix, which alters the interaction between β and the α C-terminus and thereby the relative stability of the E1P and E2P states in the catalytic cycle. Because of its low apparent K^+^ affinity, α2β2 was previously suggested to be specifically geared for high activity in astrocytes when extracellular [K^+^] is elevated, making it optimized for K^+^ clearing after neuronal bursts^16^. We suggest that another primary physiological role of the NaKA β2 is in the cerebellum, where there are only relatively few astrocytes. Future studies will be required to determine if β2 containing pumps in the cerebellar granule cells and glomeruli can compensate for the high neuronal density.

## Methods

### Chemicals

Chemicals used were obtained in the highest grade of purity from Sigma Aldrich (St. Louis, MO, USA) and VWR chemicals (Radnor, PA, USA).

### Molecular Biology

Plasmids encoding human α1, α2, α3 and β1 subunits of NaKA were purchased from Origene (Origene, Rockville, MD, USA) and subcloned into the pXOON vector^50^ using *EcoRI* and *NotI*. Plasmids encoding β2 and β3 subunits of the NaKA were purchased from Source Bioscience Lifesciences (SourceBioscience, Notingham, UK). β3 was subcloned into the pXOON vector using *EcoRI* and *XhoI*. β2 was amplified from the supplied vector using primers listed in Supplementary Table S2 introducing *HindIII* and *BamHI* restriction sites. The PCR product and pXOON were treated with *BamHI* and *HindIII* and the β2 insert was henceforth subcloned into pXOON. Chimeras were constructed by amplifying the N-terminus of β2, the transmembrane region of β2 or the N-terminus plus transmembrane region of β1 with primers listed in Supplementary Table S2 respectively. The amplified products were purified by agarose gel-electrophoresis (1% agarose (w/v)) in combination with the Qiaquick Gel Extraction Kit (Qiagen, Hilden, Germany) according to the manufacturer’s instructions. The purified product was used as a primer on pXOON β1 (β1/β2NT, β1/β2TM, β1/β2TMC) or pXOON β2 (β1/β2CT). Single mutations in wild type β1 were constructed with primers stated in supplementary table S2. All mutations were finally constructed using the quick change lightning site directed mutagenesis kit according to the manufacturer’s instructions (Agilent Technologies). Constructs were sequenced to verify successful mutagenesis. α isoforms contained mutations Q116R and N127D to reduce ouabain resistance^51^.

In preparation of mRNA transcription, the desired plasmids were linearized using *NheI* (α1, α2, α3, β1, β2 and β1/β2 mutants) or *XhoI* (β3) for 20–30 minutes. Linearized plasmids were purified using standard phenol/chloroform extraction. The restriction buffer containing the linearized plasmid and restriction enzyme was diluted to 200 μl in ddH2O and 200 μl Tris saturated phenol, pH 7.3 was added, vigorously mixed and centrifuged at approximately 17.000 x g for 2 minutes. The top fraction was mixed with chloroform and centrifuged again. After centrifugation, the top fraction was mixed with 15% (v/v) 3 M sodium acetate, pH 5.4, 2.5 volumes of ethanol and kept at –20 °C for a minimum of 30 minutes. Following that was another centrifugation at 17.000 x g and 4 °C for 30 minutes. The DNA pellet was washed with 70% (v/v) ethanol and resuspended in approximately 5–10 μl ddH2O.

mRNA was transcribed using the mMessage mMachine T7 Ultra Kit (Ambion, Life Technologies, Carlsbad, CA, USA) according to manufacturer’s instructions.

Oocytes from *Xenopus laevis* were isolated and defolliculated. 50 nl of a mixture of α (10 ng) and β (5 ng) mRNA was injected into Stage V and VI oocytes. Oocytes were incubated at 11 °C for 3–8 days prior to electrophysiological analysis.

### Electrophysiology

Electrophysiological measurements were performed with an OC-725C voltage-clamp apparatus (Warner Instruments Corp., Hamden, CT, USA) and a Digidata 1440A (Molecular Devices, Sunnyvale, CA, USA) using the two-electrode voltage-clamp technique. Measurements were performed in different buffers.

Sodium buffer (with or without 10 mM ouabain): 75.5 mM NaOH, 75.5 mM N-methyl-D-glucamin (NMDG), 110 mM succinic acid, 10 mM Hepes, 5 mM BaCl_2_, 1 mM MgCl_2_, 0.5 mM CaCl_2_, 1 μM ouabain, pH 7.4.

Potassium buffer (with or without 10 mM ouabain): 15 mM KOH, 50 mM NaOH, 50 mM NMDG, 110 mM succinic acid, 10 mM Hepes, 5 mM BaCl_2_, 1 mM MgCl_2_, 0.5 mM CaCl_2_, 1 μM ouabain, pH 7.4.

NMDG buffer (with or without 10 mM ouabain): 115 mM NMDG, 110 mM succinic acid, 10 mM Hepes, 5 mM BaCl_2_, 1 mM MgCl_2_, 0.5 mM CaCl_2_, 1 μM ouabain, pH 7.4.

NMDG/potassium buffer (with or without 10 mM ouabain): 15 mM KOH, 100 mM N-methyl-D-glucamin, 110 mM succinic acid, 10 mM Hepes, 5 mM BaCl_2_, 1 mM MgCl_2_, 0.5 mM CaCl_2_, 1 μM ouabain, pH 7.4.

Low ouabain concentrations in the measuring buffers inhibit the endogenous *Xenopus laevis* NaKA, and NMDG, which is too large a cation to be transported by the NaKA, is used to replace Na^+^. Measurements were performed in 200 ms voltage jumps in steps of 10 mV and a holding potential of –50 mV. Measurements in 10 mM ouabain buffer were subtracted from measurements without a high ouabain concentration yielding currents solely generated by the NaKA.

Charge translocation was determined by fitting single exponentials to the ouabain-sensitive pre-steady-state currents in K^+^ occluded buffer (Fig. 2): f(t) = A * exp(–t/τ) + C

where t is time after the voltage jump, A the amplitude (current at t = 0), τ the relaxation rate and C a constant (current at t = ∞).

Data was recorded and analysed with pClamp 10.4 (Molecular Devices) and Graph Pad Prism 6 (Graph Pad Software)^46^.

### Molecular Dynamics simulations

All-atom MD simulations were performed in a manner similar to one described in a recent publication^52^. The recently determined, crystal structure of the [Na_3_] E1-AlF4-ADP form of pig kidney NaKA (PDB ID: 3WGU^4^) was used as a starting point of the simulations. This crystal structure represents the pig α1β1γ ternary complex and was used as the model of the α1β1 combination of the E1P phosphoenzyme. Subsequently, this structure was embedded in a fully hydrated 1-palmitoyl,2-oleoyl-sn-glycero-3-posphocholine (POPC), including bound sodium ions and ADP, and an AlF_4_ phosphoryl transfer mimic was replaced by a fully phosphorylated Asp369 residue (the phosphorylation site^47^). To study the α1β2 and α1β3 combinations, the β1 structure was replaced by β2 and β3 homology models, respectively. For the β1/3mut and β1/β2TM combinations, the point mutations were introduced into the β1 structure using Pymol (The PyMOL Molecular Graphics System, Version 1.7.4 Schrödinger, LLC).

System construction – The Na^+^ coordinating residues E327 and E779 at binding sites I and II , as well as E954 at the unoccupied site IIIa were kept protonated^52^. The remaining glutamate and aspartate residues were kept in the charged state. The αβγ complex was embedded in the equilibrated POPC membrane (~460 lipid molecules), using the g_membed tool, and surrounded with ~60.000 water molecules. Electroneutrality was achieved by additional sodium ions, placed randomly in the aqueous solution. The resulting model was treated as the input for the MD simulations of the α1β1 combination. The atomistic homology models of the β2 and β3 subunits were constructed using the atomistic coordinates of the β1 subunit crystal structure (PDB ID: 3WGU^4^) and the human sequences of β2 and β3, respectively, using MODELLER^53^. Subsequently, the β1 subunit in the α1β1 combination was replaced by either β2, β3, β1/3mut or β1/β2TM, resulting in models that were treated as inputs for the MD simulations of the α1β2, α1β3, α1β1/3mut or α1β1/β2TM combination, respectively.

Simulation details – GROMACS version 5.0.1 was used to propagate the MD equations of motions using the leap-from algorithm. The CHARMM36 force field in GROMACS format was employed^54^,^55^,^56^ for proteins, lipids, ADP and ions. Parameters for the phosphorylated aspartate were adapted from Damjanović *et al.*^57^. Water was modelled with the CHARMM TIP3P model, with Lennard-Jones interactions between water hydrogens, which ensures the correct phase behaviour of the POPC membrane^58^. A timestep of 2 fs was used. Periodic boundary conditions were applied in all three directions. The van der Waals interactions were switched off from 0.8 to 1.2 nm, using the force-switch option in GROMACS. The Particle Mesh Ewald (PME)^59^ with a 1.2 nm cut-off was employed for electrostatic interactions. The simulated systems were maintained at the temperature of 310 K and the pressure of 1 bar, realizing the NPT statistical ensemble. Temperature coupling was realized using the Berendsen thermostat^60^ for the equilibration (10 ns) and with the Nose-Hoover thermostat^61^,^62^ for the production runs (100 ns each), separately for the solute (proteins, lipids, ADP) and the solvent (water and ions). Pressure coupling was realized with the Berendsen barostat^60^ for the equilibration, and the Parinello-Rahman barostat^63^ for the production runs. Prior to MD simulations, all systems were energy minimized with the steepest decent algorithm (10000 steps). Trajectories were sampled every 25 ps. The analysis was carried out using GROMACS suite programs: gmx bundle, gmx rms, gmx distnace, gmx rdf. Visualizations were made using VMD^64^.

### Western Blot

3 months old C57BL/6J mice (Janvier) were killed by cervical dislocation, brain regions were dissected and lysed in 10 mM Tris, 150 mM NaCl, 2 mM EDTA with 1% IGEPAL and protease inhibitor (Complete, Roche, Basel, Switzerland). Lysates were separated by SDS-PAGE and electro-blotted onto nitrocellulose membranes (Pharmacia-Amersham, Amersham, UK). Membranes were blocked in PBS with 0.5% Tween-20 and 5% skimmed milk powder and subsequently incubated with primary antibodies (β2 1:1000 (HPA010698, Atlas Antibodies, Stockholm, Sweden) and GAPDH 1:1000 (ab9485, Abcam, Cambridge, UK)) overnight at 4 °C. The following day, membranes were incubated with horseradish peroxidase-conjugated secondary antibody (pig anti-rabbit 1:2000 (Dako, Glostrup, Denmark)) for 1 hour at room temperature. Visualization was done using a LAS 3000 imager (Fujifilm, Tokyo, Japan) with Amersham ECL Western Blotting Detection Kit (GE Healthcare, Buckinghamshire, UK) as detection reagent.

### Fluorescence immunohistochemistry

3 months old C57BL/6J mice (Janvier) were anesthetized by intraperitoneal injection of 250 mg/kg pentobarbital (Mebumal SAD, Copenhagen, Denmark) and afterwards transcardially perfused by 25 ml *Phosphate-buffered saline* (PBS) (10 mM PO_4_^3−^, 137 mM NaCl, and 2.7 mM KCl), followed by 25 ml 4% paraformaldehyde in PBS. Brains were dissected, post fixated in 4% paraformaldehyde in PBS overnight at 4 °C and stored in sucrose solution (25% w/v sucrose, in PBS) at 4 °C. Brains were cut on a HM450 sledge microtome (Microm International, Walldorf, Germany) as 40 μm coronal sections that were stored in cryoprotectant (30% ethylene glycol, 26% glycerol, in PBS) at –20 °C.

For staining, stored sections were washed in PBS pH 7.4 and blocked in 5% donkey serum PBS/Triton X-100 0.25% for 1 hour at RT. Primary antibodies (α1 (1:200) (a6f-c, Developmenal Studies Hybridoma Bank), β1 (1:100) (ABN722, Merck Millipore, Darmstadt, Germany), β2 (1:50) (HPA010698, Merck Millipore, Darmstadt, Germany), NeuN (1:300) (MAB377B, Merck Millipore, Darmstadt, Germany))^22^ were applied in 1% donkey serum PBS/Triton X-100 0.25% for 1 hour at RT and then overnight at 4 °C. The following day secondary labelling was done with Alexa Fluor fluorescent-conjugated secondary antibodies (Alexa Fluor 488 donkey anti rabbit (A21206, Life Technologies, Carlsbad, CA, USA), Alexa Fluor 568 donkey anti mouse (A10037, Life Technologies, Carlsbad, CA, USA), Alexa Fluor 568 Streptavidin (S11226, Life Technologies, Carlsbad, CA, USA)) (1:350) in 1% donkey serum PBS/Triton X-100 0.25% for 1 hour at RT. Hoechst (1:10000) (Life technologies, Carlsbad, CA, USA) in PBS was used to counterstain the nuclei. Sections were mounted using fluorescence mounting medium (Dako, Glostrup, Denmark) and analysed on a LSM510 laser-scanning confocal microscope using a 40x and 63x C-Apochromat water immersion objective NA 1.2 (Carl Zeiss, Göttingen, Germany). Zen 2011 software (Carl Zeiss, Göttingen, Germany) was used for image capturing and subsequent image analysis.

## Additional Information

**How to cite this article**: Hilbers, F. *et al.* Tuning of the Na,K-ATPase by the beta subunit. *Sci. Rep.* **6**, 20442; doi: 10.1038/srep20442 (2016).

## Supplementary Material

Supplementary Information

## References

[b1] Morth J. P. *et al.* Crystal structure of the sodium-potassium pump. Nature 450, 1043–1049 (2007). 18075585 10.1038/nature06419

[b2] Shinoda T., Ogawa H., Cornelius F. & Toyoshima C. Crytal Structure of the sodium-potassium pump at 2.4A resolution. Nature 459, 446–451 (2009). 19458722 10.1038/nature07939

[b3] Toyoshima C., Kanai R. & Cornelius F. First Crystal Structures of Na^+^,K^+^-ATPase: New Light on the Oldest Ion Pump. Structure 19, 1732–1738 (2011). 22153495 10.1016/j.str.2011.10.016

[b4] Kanai R., Ogawa H., Vilsen B., Cornelius F. & Toyoshima C. Crystal structure of a Na^+^-bound Na^+^,K^+^-ATPase preceding the E1P state. Nature 502, 201–6 (2013). 24089211 10.1038/nature12578

[b5] Nyblom M. *et al.* Crystal Structure of Na^+^, K^+^-ATPase in the Na^+^-Bound State. Science (80-.). 342, 123–127 (2013). 10.1126/science.1243352 24051246

[b6] Laursen M., Yatime L., Nissen P. & Fedosova N. U. Crystal structure of the high-affinity Na^+^, K^+^-ATPase - ouabain complex with Mg2^+^ bound in the cation binding site. Proc. Natl. Acad. Sci. USA. 110, 10958–10963 (2013). 23776223 10.1073/pnas.1222308110 PMC3704003

[b7] Albers R. W. Biochemical aspects of active transport. Annu. Rev. Biochem. 36, 727–756 (1967). 18257736 10.1146/annurev.bi.36.070167.003455

[b8] Post R. L., Kume S., Tobin T., Orcutt B. & Sen A. K. Flexibility of an active center in sodium-plus-potassium adenosine triphosphatase. J. Gen. Physiol. 54, 306–326 (1969). 19873651 10.1085/jgp.54.1.306 PMC2225916

[b9] Holmgren M. *et al.* Three distinct and sequential steps in the release of sodium ions by the Na^+^/K^+^-ATPase. Nature 403, 898–901 (2000). 10706288 10.1038/35002599

[b10] Garrahan P. J. & Glynn I. M. The incorporation of inorganic phosphate into adenosine triphosphate by reversal of the sodium pump. J Physiol 192, 237–256 (1967). 4228076 10.1113/jphysiol.1967.sp008298 PMC1365483

[b11] Tokhtaeva E., Clifford R. J., Kaplan J. H., Sachs G. & Vagin O. Subunit isoform selectivity in assembly of Na,K-ATPase α-β heterodimers. J. Biol. Chem. 287, 26115–26125 (2012). 22696220 10.1074/jbc.M112.370734 PMC3406695

[b12] Roy M., Sivan-Loukianova E. & Eberl D. F. Cell-type-specific roles of Na^+^/K^+^ ATPase subunits in Drosophila auditory mechanosensation. Proc. Natl. Acad. Sci. USA. 110, 181–6 (2013). 23248276 10.1073/pnas.1208866110 PMC3538205

[b13] Magyar J. P. *et al.* Degeneration of neural cells in the central nervous system of mice deficient in the gene for the adhesion molecule on glia, the β2 subunit of murine Na,K-ATPase. J. Cell Biol. 127, 835–845 (1994). 7525597 10.1083/jcb.127.3.835 PMC2120225

[b14] Senner V. *et al.* AMOG/β2 and glioma invasion: Does loss of AMOG make tumour cells run amok? Neuropathol. Appl. Neurobiol. 29, 370–377 (2003). 12887597 10.1046/j.1365-2990.2003.00473.x

[b15] Crambert G. Transport and Pharmacological Properties of Nine Different Human Na,K-ATPase Isozymes. J. Biol. Chem. 275, 1976–1986 (2000). 10636900 10.1074/jbc.275.3.1976

[b16] Larsen B. R. *et al.* Contributions of the Na^+^/K^+^-ATPase, NKCC1, and Kir4.1 to hippocampal K^+^ clearance and volume responses. Glia 62, 608–622 (2014). 24482245 10.1002/glia.22629 PMC4302754

[b17] DiFranco M., Hakimjavadi H., Lingrel J. B. & Heiny J. a. Na,K-ATPase 2 activity in mammalian skeletal muscle T-tubules is acutely stimulated by extracellular K^+^. J. Gen. Physiol. 146, 281–294 (2015). 26371210 10.1085/jgp.201511407 PMC4586590

[b18] Zhang L., Morris K. J. & Ng Y.-C. Fiber type-specific immunostaining of the Na^+^,K^+^-ATPase subunit isoforms in skeletal muscle: Age-associated differential changes. Biochim. Biophys. Acta - Mol. Basis Dis. 1762, 783–793 (2006). 10.1016/j.bbadis.2006.08.006 PMC1761903 16979878

[b19] Pagliusi S. *et al.* Identification of a cDNA Clone Specific for the Neural Cell Adhesion Molecule AMOG. J. Neurosci. Reasearch 22, 113–119 (1989). 10.1002/jnr.490220202 2468782

[b20] Peng L., Martin-Vasallo P. & Sweadner K. J. Isoforms of Na,K-ATPase alpha and beta subunits in the rat cerebellum and in granule cell cultures. J. Neurosci. 17, 3488–3502 (1997). 9133374 10.1523/JNEUROSCI.17-10-03488.1997 PMC6573685

[b21] Howarth C., Gleeson P. & Attwell D. Updated energy budgets for neural computation in the neocortex and cerebellum. J. Cereb. Blood Flow Metab. 32, 1222–1232 (2012). 22434069 10.1038/jcbfm.2012.35 PMC3390818

[b22] Mullen R. J., Buck C. R. & Smith A. M. NeuN, a neuronal specific nuclear protein in vertebrates. Development 116, 201–211 (1992). 1483388 10.1242/dev.116.1.201

[b23] Colina C. *et al.* Structural basis of Na^(+)^/K^(+)^-ATPase adaptation to marine environments. Nat. Struct. Mol. Biol. 14, 427–431 (2007). 17460695 10.1038/nsmb1237

[b24] Poulsen H. *et al.* Neurological disease mutations compromise a C-terminal ion pathway in the Na^(+)^/K^(+)^-ATPase. Nature 467, 99–102 (2010). 20720542 10.1038/nature09309

[b25] Rakowski R. F., Vasilets L. A., LaTona J. & Schwarz W. A negative slope in the current-voltage relationship of the Na^+^/K^+^ pump in Xenopus oocytes produced by reduction of external [K^+^]. J. Membr. Biol. 121, 177–187 (1991). 1880791 10.1007/BF01870531

[b26] Efthymiadis A., Rettinger J. & Schwarz W. Inward-directed current generated by the Na^+^,K^+^ pump in Na^+^ and K^+^ free medium. Cell Biol. Int. 17, 1107–1116 (1993). 8118453 10.1006/cbir.1993.1043

[b27] Glynn I. M. & Karlish S. J. ATP hydrolysis associated with an uncoupled sodium flux through the sodium pump: evidence for allosteric effects of intracellular ATP and extracellular sodium. J Physiol 256, 465–496 (1976). 16992511 10.1113/jphysiol.1976.sp011333 PMC1309316

[b28] Heyse S., Wuddel I., Apell H. J. & Stürmer W. Partial reactions of the Na, K-ATPase: determination of rate constants. J. Gen. … 104, (1994). 10.1085/jgp.104.2.197 PMC2229205 7807047

[b29] Poulsen H. *et al.* Neurological disease mutations compromise a C-terminal ion pathway in the Na^(+)^/K^(+)^-ATPase. Nature 467, 99–102 (2010). 20720542 10.1038/nature09309

[b30] Meier S., Tavraz N. N., Dürr K. L. & Friedrich T. Hyperpolarization-activated inward leakage currents caused by deletion or mutation of carboxy-terminal tyrosines of the Na^+^/K^+^-ATPase {alpha} subunit. J. Gen. Physiol. 135, 115–134 (2010). 20100892 10.1085/jgp.200910301 PMC2812498

[b31] Azevedo F. A. C. *et al.* Equal numbers of neuronal and nonneuronal cells make the human brain an isometrically scaled-up primate brain. J. Comp. Neurol. 513, 532–541 (2009). 19226510 10.1002/cne.21974

[b32] Hasler U., Greasley P. J., Von Heijne G. & Geering K. Determinants of topogenesis and glycosylation of type II membrane proteins. Analysis of Na,K-ATPase β1 and β3 subunits by glycosylation mapping. J. Biol. Chem. 275, 29011–29022 (2000). 10887183 10.1074/jbc.M002867200

[b33] Kaplan J. H. Biochemistry of Na,K-ATPase. Annu. Rev. Biochem. 71, 511–535 (2002). 12045105 10.1146/annurev.biochem.71.102201.141218

[b34] Jaunin P. *et al.* Role of the transmembrane and extracytoplasmic domain of β subunits in subunit assembly, intracellular transport, and functional expression of Na,K-pumps. J. Cell Biol. 123, 1751–1759 (1993). 8276895 10.1083/jcb.123.6.1751 PMC2290884

[b35] Geering K. Functional roles of Na,K-ATPase subunits. Curr. Opin. Nephrol. Hypertens. 17, 526–532 (2008). 18695395 10.1097/MNH.0b013e3283036cbf

[b36] Hasler U. *et al.* Role of β-subunit domains in the assembly, stable expression, intracellular routing, and functional properties of Na,K-ATPase. J. Biol. Chem. 273, 30826–30835 (1998). 9804861 10.1074/jbc.273.46.30826

[b37] Beggah A. T., Jaunin P. & Geering K. Role of glycosylation and disulfide bond formation in the β subunit in the folding and functional expression of Na,K-ATPase. J. Biol. Chem. 272, 10318–10326 (1997). 9092584 10.1074/jbc.272.15.10318

[b38] Jaisser F., Jaunin P., Geering K., Rossier B. C. & Horisberger J. D. Modulation of the Na,K-pump function by beta subunit isoforms. J. Gen. Physiol. 103, 605–623 (1994). 8057080 10.1085/jgp.103.4.605 PMC2216863

[b39] Chow D. a R. C. & Forte J. G. Functional significance of the beta subunit for heterodimeric P-Type ATPases. J. Exp. Biol. 198, 1–17 (1995). 7891030 10.1242/jeb.198.1.1

[b40] Geering K. *et al.* Oligomerization and maturation of Na^+^,K^+^-ATPase: functional interaction of the cytoplasmic NH2-terminus of the beta subunit with the alpha subunit. J. Cell Biol. 133, 1193–1204 (1996). 8682858 10.1083/jcb.133.6.1193 PMC2120891

[b41] Thompson C. B. & McDonough A. a. Skeletal muscle Na,K-ATPase α and β subunit protein levels respond to hypokalemic challenge with isoform and muscle type specificity. J. Biol. Chem. 271, 32653–32658 (1996). 8955095 10.1074/jbc.271.51.32653

[b42] Paulusma C. C. & Oude Elferink R. P. J. P4 ATPases - The physiological relevance of lipid flipping transporters. FEBS Letters 584, 2708–2716 (2010). 20450914 10.1016/j.febslet.2010.04.071

[b43] Lutsenko S. & Kaplan J. H. Evidence of a role for the Na,K-ATPase beta-subunit in active cation transport. Ann. N. Y. Acad. Sci. 671, 147–154; discussion 154–155 (1992). 1337669 10.1111/j.1749-6632.1992.tb43792.x

[b44] Liu C. C. *et al.* Redox-dependent regulation of the Na^+^-K^+^ pump: New twists to an old target for treatment of heart failure. Journal of Molecular and Cellular Cardiology 61, 94–101 (2013). 23727392 10.1016/j.yjmcc.2013.05.013

[b45] Toustrup-Jensen M. S. *et al.* The C terminus of Na^+^,K^+^-ATPase controls Na^+^ affinity on both sides of the membrane through Arg935. J. Biol. Chem. 284, 18715–18725 (2009). 19416970 10.1074/jbc.M109.015099 PMC2707196

[b46] Paulsen P. A. *et al.* The C-terminal cavity of the Na,K-ATPase analyzed by docking and electrophysiology. Mol. Membr. Biol. 30, 1–11 (2012). 10.3109/09687688.2012.713520 22913437

[b47] Olesen C. *et al.* The structural basis of calcium transport by the calcium pump. Nature 450, 1036–1042 (2007). 18075584 10.1038/nature06418

[b48] Bublitz M. *et al.* Ion pathways in the sarcoplasmic reticulum Ca2^+^-ATPase. Journal of Biological Chemistry 288, 10759–10765 (2013). 23400778 10.1074/jbc.R112.436550 PMC3624456

[b49] Olesen C. *et al.* The structural basis of calcium transport by the calcium pump. Nature 450, 1036–1042 (2007). 18075584 10.1038/nature06418

[b50] Jespersen T., Grunnet M., Angelo K., Klaerke D. A. & Olesen S. P. Dual-function vector for protein expression in both mammalian cells and Xenopus laevis oocytes. BioTechniques 32, (2002). 10.2144/02323st05 11911656

[b51] Price E. M. & Lingrel J. B. Structure-function relationships in the Na,K-ATPase alpha subunit: site-directed mutagenesis of glutamine-111 to arginine and asparagine-122 to aspartic acid generates a ouabain-resistant enzyme. Biochemistry 27, 8400–8408 (1988). 2853965 10.1021/bi00422a016

[b52] Mahmmoud Y. a., Kopec W. & Khandelia H. K^+^ Congeners That Do not Compromise Na^+^ Activation of the Na ^+^, K ^+^ -ATPase. Hydration of the Ion binding Cavity Likely Controls Ion Selectivity. J. Biol. Chem. 290, 3720–3730 (2014). 25533461 10.1074/jbc.M114.577486 PMC4319036

[b53] Fiser A. & Šali A. MODELLER: Generation and Refinement of Homology-Based Protein Structure Models. Methods in Enzymology 374, 461–491 (2003). 14696385 10.1016/S0076-6879(03)74020-8

[b54] Mackerell A. D. Empirical force fields for biological macromolecules: Overview and issues. Journal of Computational Chemistry 25, 1584–1604 (2004). 15264253 10.1002/jcc.20082

[b55] Klauda J. B. *et al.* Update of the CHARMM All-Atom Additive Force Field for Lipids: Validation on Six Lipid Types. J. Phys. Chem. B 114, 7830–7843 (2010). 20496934 10.1021/jp101759q PMC2922408

[b56] MacKerell a D. *et al.* All-atom empirical potential for molecular modeling and dynamics studies of proteins. J. Phys. Chem. B 102, 3586–616 (1998). 24889800 10.1021/jp973084f

[b57] Damjanović A., García-Moreno E., B. & Brooks B. R. Self-guided Langevin dynamics study of regulatory interactions in NtrC. Proteins Struct. Funct. Bioinforma. 76, 1007–1019 (2009). 10.1002/prot.22439 PMC3373014 19384996

[b58] Piggot T. J., Piñeiro Á. & Khalid S. Molecular dynamics simulations of phosphatidylcholine membranes: A comparative force field study. J. Chem. Theory Comput. 8, 4593–4609 (2012). 26605617 10.1021/ct3003157

[b59] Darden T., York D. & Pedersen L. Particle mesh Ewald: An N log(N) method for Ewald sums in large systems. J. Chem. Phys. 98, 10089 (1993).

[b60] Berendsen H. J. C., Postma J. P. M., van Gunsteren W. F., DiNola a. & Haak & J. R. Molecular dynamics with coupling to an external bath. J. Chem. Phys. 81, 3684–3690 (1984).

[b61] Nose S. A unified formulation of the constant temperature molecular dynamics methods. J. Chem. Phys. 81, 511–519 (1984).

[b62] Hoover W. G. Canonical dynamics: Equilibrium phase-space distributions. Phys. Rev. A 31, 1695–1697 (1985). 9895674 10.1103/physreva.31.1695

[b63] Parrinello M. & Rahman a. Polymorphic Transitions in Single Crystals: a New Molecular Dynamics Method. J. Appl. Phys. 52, 7182–7190 (1981).

[b64] Humphrey W., Dalke A. & Schulten K. VMD: Visual molecular dynamics. J. Mol. Graph. 14, 33–38 (1996). 8744570 10.1016/0263-7855(96)00018-5

