# Supplementary material for: Tuning of the Na,K-ATPase by the beta subunit
Source: Sci Rep. 2016 Feb 5;6:20442. doi: 10.1038/srep20442 (PMC4742777; doi:10.1038/srep20442)
Supplement: Supplementary Information [file srep20442-s1.pdf]

Supplementary Information for

Tuning of the Na,K-ATPase by the beta subunit

Florian Hilbers[1,2,3], Wojciech Kopec[4], Toke Jost Isaksen[3,5], Thomas Hellesøe Holm[3,5], Karin Lykke-Hartmann[3,5,6], Poul Nissen[1,2,3], Himanshu Khandelia[4] and Hanne Poulsen[1,2,3,*]

[1]Danish Research Institute of Translational Neuroscience – DANDRITE, Nordic-EMBL Partnership for Molecular Medicine, Aarhus University, DK-8000 Aarhus, Denmark

[2]Department of Molecular Biology and Genetics, Aarhus University, DK-8000 Aarhus, Denmark

[3]Centre for Membrane Pumps in Cells and Disease – PUMPKIN, Danish National Research Foundation, DK-8000 Aarhus, Denmark

[4]MEMPHYS: Centre for Biomembrane Physics, University of Southern Denmark, DK-8000 Aarhus, Denmark

[5]Department of Biomedicine, Aarhus University, DK-8000 Aarhus, Denmark

[6]Aarhus Institute of Advanced Studies (AIAS), Aarhus University, DK-8000 Aarhus, Denmark

[*]corresponding author

Contact: hp@mb.au.dk

Supplementary Table S1: Fit-Parameters of charge translocation curves, rate constants at -140 mV and tilt angle predictions from MD simulations. Data are represented as mean ± SD, n.d.: not determined.

Construct	***Midpoint / mV***	***Valence***	***Rate constant / s-1***	***Tilt angle (MD)***
α1β1	-82.8 ± 0.6	0.796 ±0.01	208.4 ± 2.1	32.7° ± 1.8°
α1β2	-29.4 ± 0.4	0.907 ± 0.01	343.7 ± 43.9	38.0° ± 0.7°
α1β3	-69.8 ± 1.3	0.851 ± 0.03	197.7 ± 8.8	31.3° ± 0.9°
α2β1	-18.1 ± 0.9	0.842 ± 0.03	400.1 ± 25.1	n.d.
α2β2	14.4 ± 1.2	0.948 ± 0.03	641.6 ± 74.6	n.d.
α2β3	-17.7 ± 1.6	0.781 ± 0.04	497.1 ± 29.8	n.d.
α3β1	-59.7 ± 0.9	0.735 ± 0.02	210.6 ± 38.9	n.d.
α3β2	-28.7 ± 1.1	0.869 ± 0.03	385.8 ± 12.4	n.d.
α3β3	-54.3 ± 1.3	0.775 ± 0.03	205.8 ± 32.8	n.d.
α1β1/β2NT	-80.2 ± 0.2	0.789 ± 0.01	174.6 ± 16	n.d.
α1β1/β2TM	-51.4 ± 0.4	0.847 ± 0.01	240.9 ± 42	37.2° ± 0.7°
α1β1/β2CT	-70.4 ± 0.3	0.837 ± 0.01	203.5 ± 95	n.d.
α1β1/β2TMC	-86.2 ± 1.4	0.756 ± 0.03	220.3 ± 56	n.d.
α1β1AF	-77.9 ±0.8	0.852 ± 0.02	152.5 ± 31	n.d.
α1β1TAM	-75.4 ± 0.5	0.857 ± 0.01	140.2 ± 24	n.d.
α1β1/3mut	-64.7 ± 0.8	0.889 ± 0.03	203.8 ± 28.5	36.0° ± 0.8°

Supplementary Table S2: Primers used to construct the β1-β2 chimeras and β1 mutants.

Primer	***Sequence***
β2 fwd	GTGCAAGGATCCAACGAATTCGCCCTT
β2 rev	GAATTGAATTTAGCGGAAGCTTGATGCC
β1/β2NT fwd	CAGCTGCTGACCCGCCATCGCCATGGTCATCCAGAAAGA
β1/β2NT rev	AGAAGGATCTTAAACCAACTGCCACCGGTGCGGCCCATAAACTG
β1/β2TM fwd	AGGAGTTTCTGGGCAGGACCGGTGGCAGCTGGGCCTTTATCCTC
β1/β2TM rev	ATCCTGGCGGGGCCACTCGGTCCTGATATGTGGGGGTATGGTCGGA
β1/β2CT fwd	GCCCGGACTCGCCCCGCGCCACCAAGATGGCCCGCGGGAAAGCCAAG
β1/β2CT rev	GGCCAGTCGGTCCTGGTACTTGGGCTTAAATTCACTATGGTGA
β1/β2TMC fwd	TATGGCTGCCTGGCTGGCATCTTCACCCTCACCATGTGGGT
β1 VSD fwd	CTGATATGTGGGCTTAAAATCACTGACGGTGAGCAGCATCACTTG
β1 VSD rev	CAAGTGATGCTGCTCACCGTCAGTGATTTTAAGCCCACATATCAG
β1 AF fwd	AAAATATTACGTAGAATAGAAGGATGAAAGCCCAACTGCCACCGGTCCTGCCCAG
β1 AF rev	CTGGGCAGGACCGGTGGCAGTTGGGCTTTCATCCTTCTATTCTACGTAATATTTT
β1 TAM fwd	TGGCTGCCTGACTGCCATGTTCATCGGAACCATCCA
β1 TAM rev	TGGTTCCGATGAACATGGCAGTCAGGCAGCCATAAAATAT

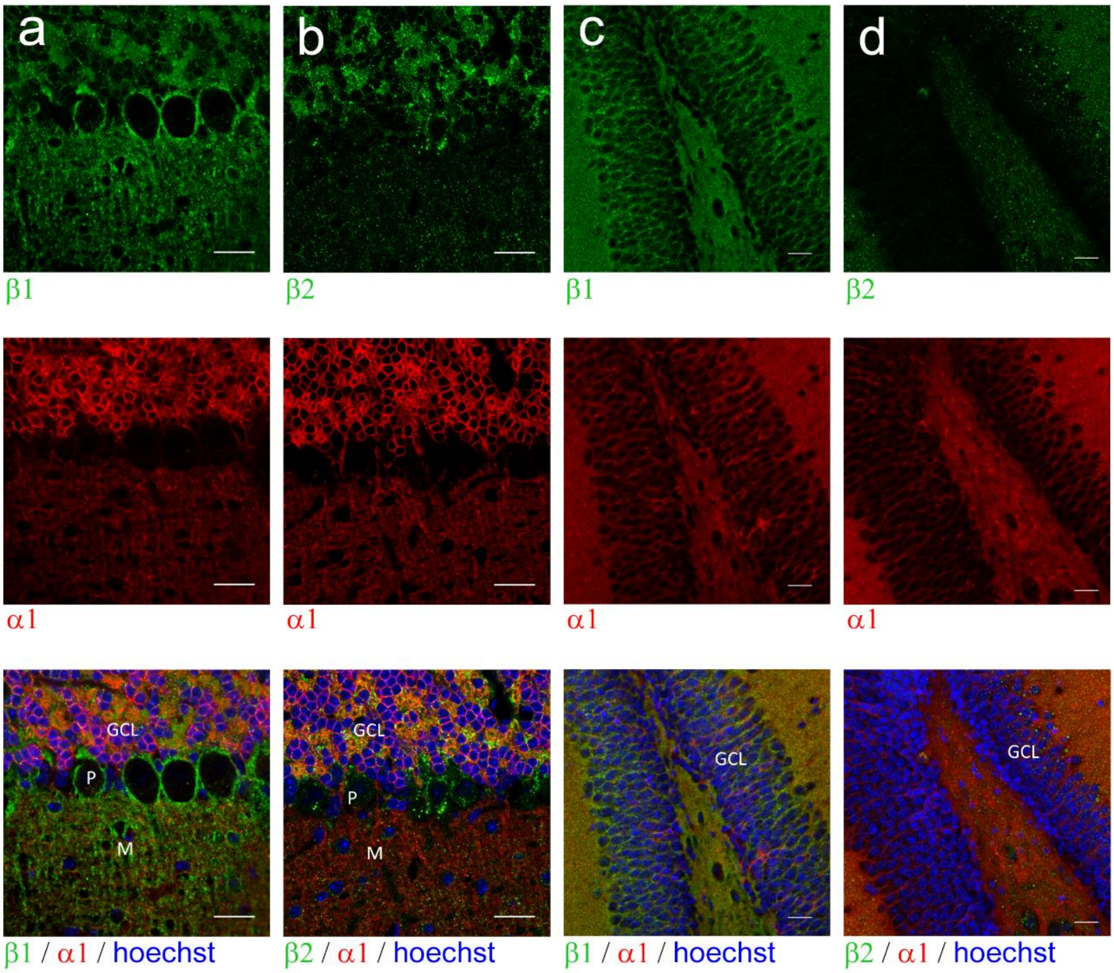

Supplementary Figure S1: Localization of α1, β1 and β2 subunit isoforms in cerebellum and dentate gyrus in brain slices from three month old mice.

a-b) Fluorescence immunohistochemistry of β1 and β2 in cerebellum co-stained with α1.

c-d) β1 and β2 in the dentate gyrus of hippocampus co-stained with α1. GCL: granule cell layer. P: Purkinje cell layer. ML: molecular layer. Scale bars represent 20 µm.

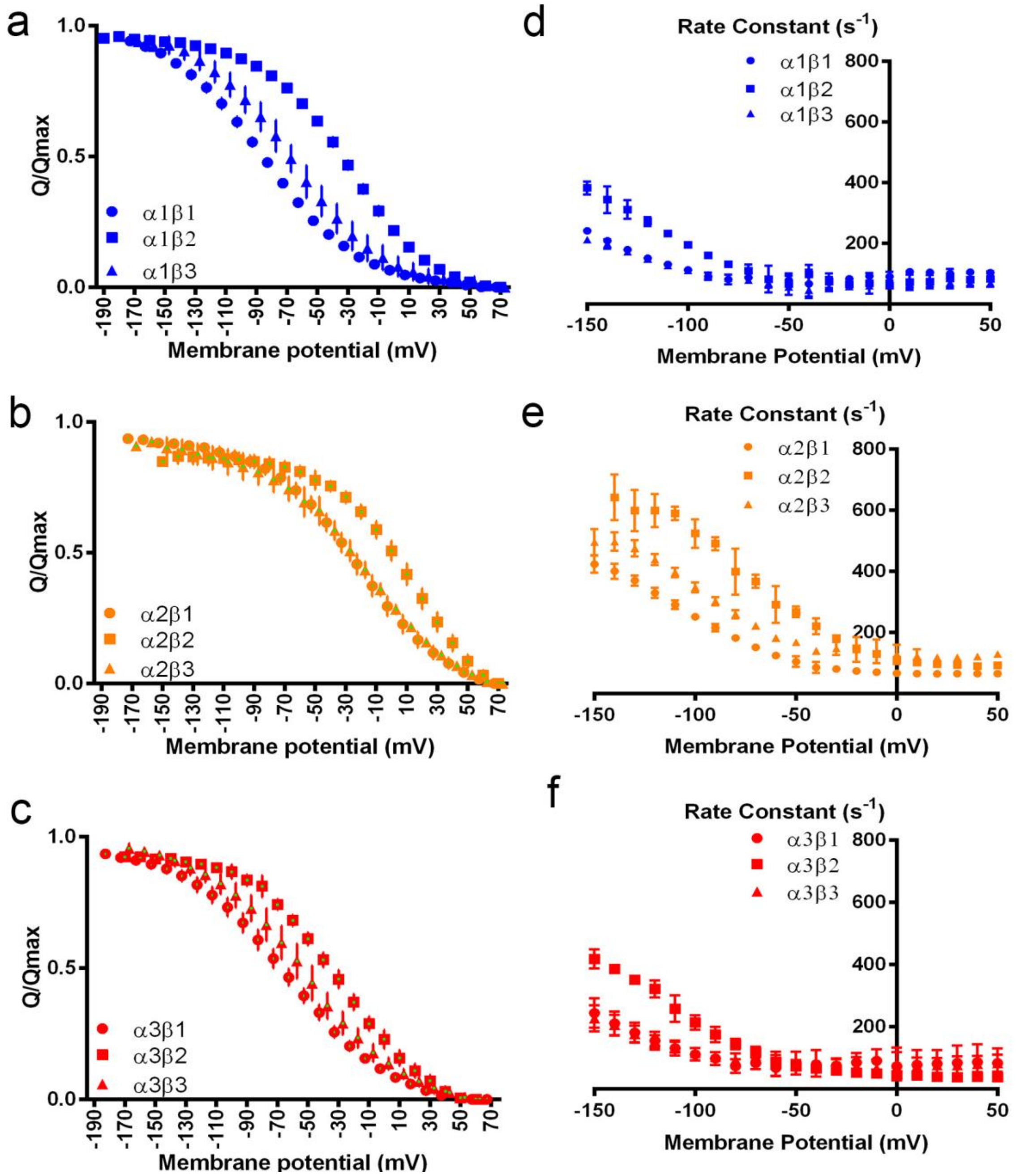

Supplementary Figure S2: Voltage-dependence of charge translocation and rate constants of nine human αβ combinations.

Three human α isoforms were paired with the three β isoforms. The difference curves in K^+-free buffers with and without 10 mM ouabain were fitted with single exponentials and analyzed as described in Fig.1 yielding the charge translocation curves a), b), c) and on-rate constants d), e) and f) for α1, α2 and α3 respectively.

N = 3-8 with oocytes from at least two *Xenopus laevis* females. Data are represented as mean ± SD.

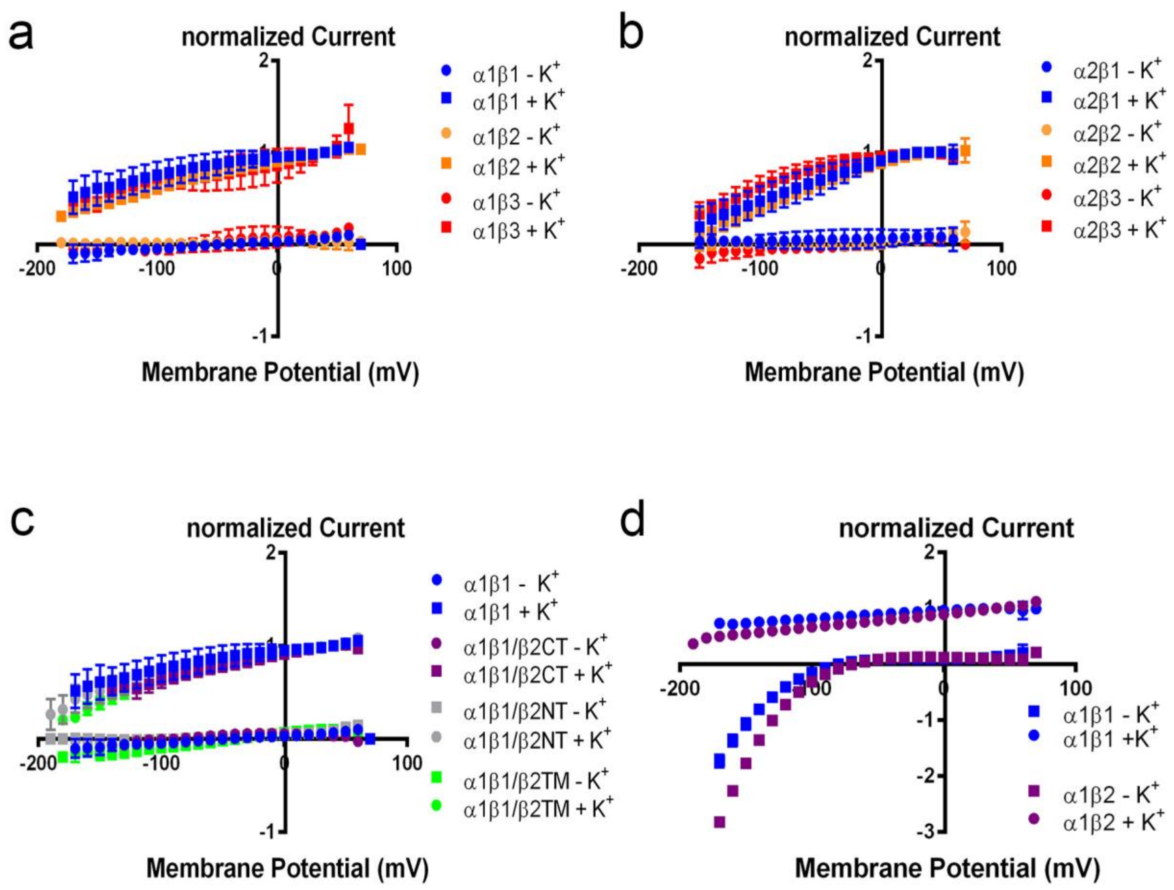

Supplementary Figure S3: Voltage dependence of steady-state currents for six human αβ combinations and three chimeras.

The forward ouabain-sensitive current induced by 15 mM K^+ is similar for a) α1, b) α2 and c) the chimeras.

d) In the absence of extracellular Na^+, the forward K^+-induced, ouabain-sensitive currents are similar with α1β1 and α1β2. In the absence of K^+ and Na^+, both combinations have inwardly rectifying currents [20], slightly more pronounced for β2.

N = 3 with oocytes from at least 2 *Xenopus laevis* females. Data are represented as mean ± SD.

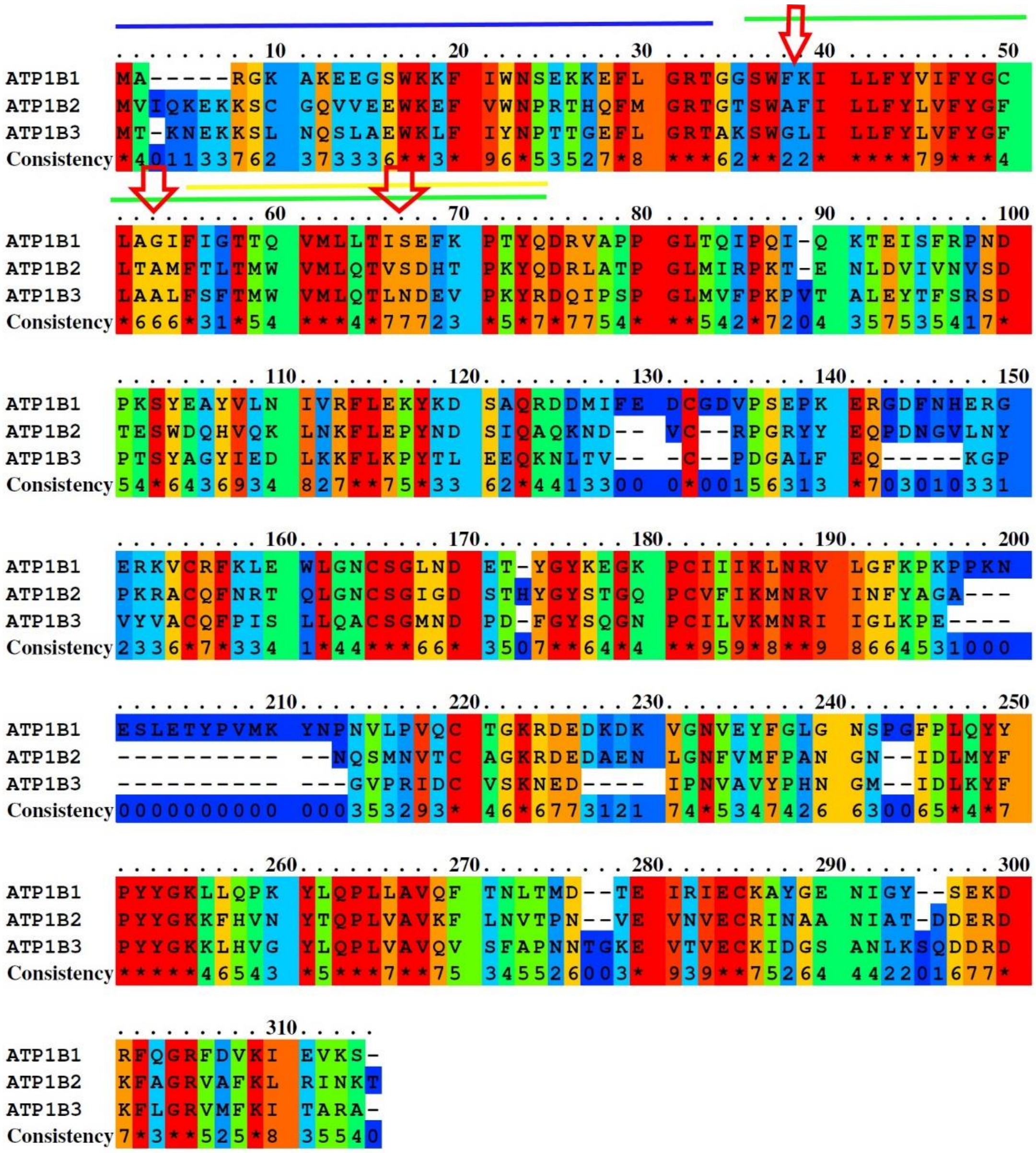

Supplementary Figure S4: Sequence comparison of the three human β isoforms.

The dark blue bar above the numbering denotes the area of β1 that was exchanged for the equivalent area of β2 to yield β1/β2NT, the green bar shows the area of β1 that was exchanged for that of β2 to yield β1/β2TM, and the yellow bar denotes the area swapped to yield β1/β2TMC. The unmarked area is the C-terminal domain, where the β1 sequence was swapped with that of β2 to yield β1/β2CT. The FK, AGI and VSD pockets are denoted by arrows. Fully conserved residues are shown in red. The lower the consistency score, the less conserved the residues. Figure was made using the PRALINE software server.

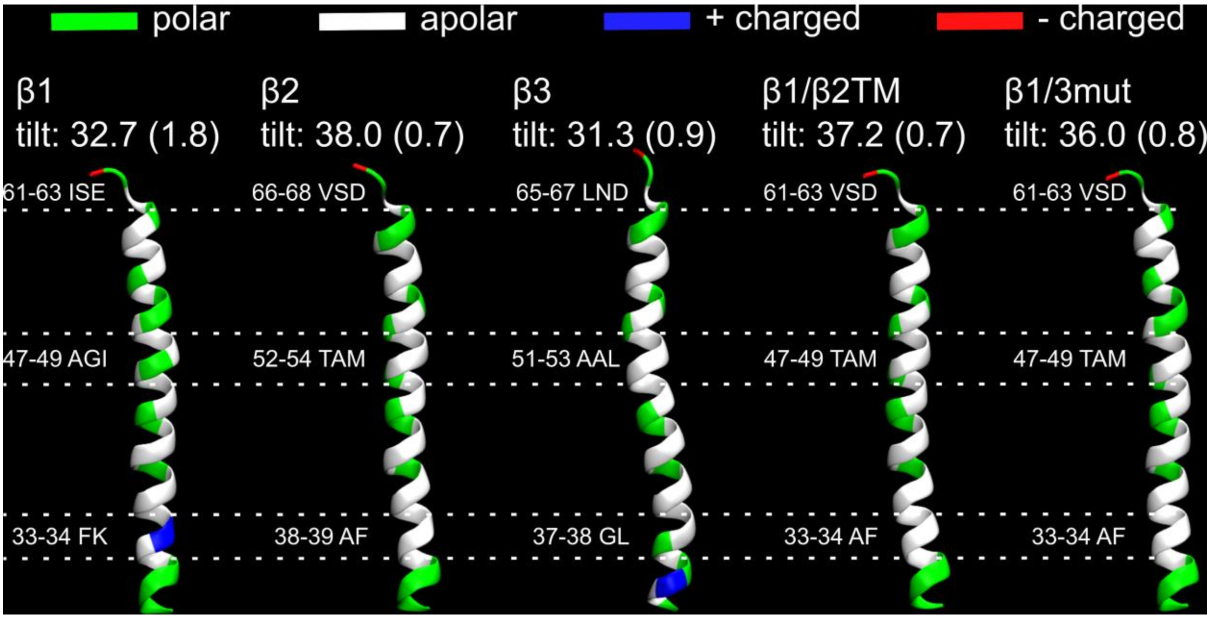

Supplementary Figure S5: Comparison of the β transmembrane helices in MD simulations of α1β1, α1β2, α1β3, α1β1/β2TM and α1β1/3mut.

The β transmembrane helices alone are shown from MD simulations of the indicated full-length β in complex with α1 and the γ subunit in a POPC lipid membrane. The 33-34 and 61-63 residues located at or near the membrane interfaces as well as the central 47-49 residues are indicated with dotted lines (β1 numbering, corresponding residues in β2 and β3 are labelled similarly). The helix tilt is given for each β helix, defined as the angle between the helix axis and the z-axis perpendicular to the membrane surface. Presented values are the averages from the last 40 ns of simulations, with error estimations obtained with block averaging. Polar, apolar and charged residues are color coded as indicated.

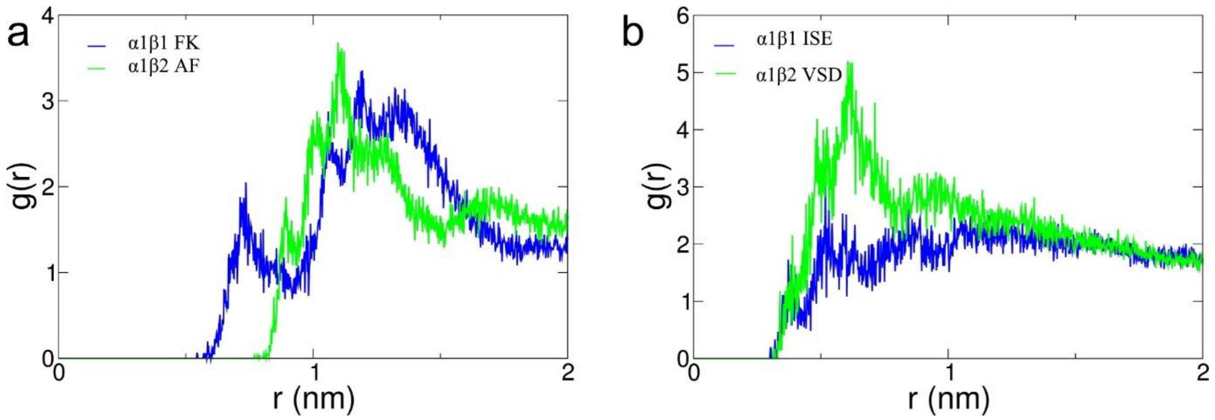

Supplementary Figure S6: Radial distribution functions (RDFs) for the residues that anchor the transmembrane helix of the β1 and β2 subunits in the lipid bilayer and the lipid headgroups.

a) Interactions between the POPC headgroups and the N-terminus of the β transmembrane helix (residues 33-34, β1 numbering). The interactions of residues from β1 are plotted in blue, from β2 in green. The last 40 ns of the simulations were used to calculate RDFs.

b) Interactions between the POPC headgroups and the C-terminus of the β transmembrane helix (residues 61-63, β1 numbering).
